# A case of ankylosing spondylitis presenting with fever of unknown origin diagnosed as aortitis: A case report

**DOI:** 10.1002/ccr3.8207

**Published:** 2023-11-16

**Authors:** Mahsa Mehdipour Dalivand, Rezvan Abdolazimi, Reyhaneh Manafi‐Farid, Ahmadreza Jamshidi, Kimia Kassaee, Sara Foolad, Majid Alikhani

**Affiliations:** ^1^ Rheumatology Research Center, Shariati Hospital Tehran University of Medical Sciences Tehran Iran; ^2^ Department of Internal Medicine, Shariati Hospital Tehran University of Medical Sciences Tehran Iran; ^3^ Research Center for Nuclear Medicine, Shariati Hospital Tehran University of Medical Sciences Tehran Iran; ^4^ School of Medicine Iran University of Medical Sciences Tehran Iran; ^5^ College of Agricultural Sciences, Shiraz Branch Islamic Azad University Shiraz Iran

**Keywords:** ankylosing, aortitis, arthralgia, case report, spondylitis, spondyloarthritis

## Abstract

**Key Clinical Message:**

Clinicians should be aware of rare manifestations of AS, while considering a low threshold for screening vascular involvement in an axial SpA/nrxSpA/AS presenting with unexplained fevers and significant constitutional symptoms and elevated markers.

**Abstract:**

Ankylosing spondylitis (AS) is a chronic inflammatory disease from the spondyloarthritis complex, which usually affects young men and primarily involves sacroiliac joints and the spine. It can also present with non‐joint involvement, such as cardiovascular manifestations. Aortitis is a rare yet critical cardiovascular complication associated with AS, which can lead to life‐threatening outcomes when undiagnosed. Here we report a 34‐year‐old man with intermittent fevers and significant weight loss, myalgia, and arthralgia for 1 year before being referred to our hospital due to undefinable causes despite multiple diagnostic efforts. The patient presented with elevated inflammatory markers and involvement of sacroiliac joints in favor of the AS. A positron emission tomography scan was also done to rule out underlying malignancy, which led to the detection of inflammation in ascending aorta, compatible with aortitis. The patient was treated with nonsteroidal anti‐inflammatory drugs, prednisolone, and infliximab, and his signs and symptoms significantly improved. Our case reports a rare but substantial complication of AS, in a young patient without a history of prolonged disease presenting with unspecific manifestations. The implantation of a thorough examination of AS patients, including cardiac examinations, could contribute to faster and more efficient diagnosis and treatment.

## Graphical abstract

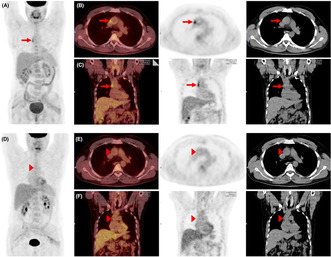

## 1 INTRODUCTION

Ankylosing Spondylitis (AS) is a chronic inflammatory rheumatic disease strongly associated with HLA B27, usually starting in the third decade of life and mostly seen in men.^1^, ^2^, ^3^ It mainly affects the sacroiliac and axial joints, but other organs, such as the eyes, heart, kidneys, and lungs, have also been reported to be involved. Cardiac involvement, including aortitis, aortic valve insufficiency, and cardiac conductive disorders manifests in 2%–10% of patients, increasing the morbidity of patients with AS.^4^ Furthermore, the prevalence of aortic root or valve disease and conduction disturbances in spondyloarthritides has been described in 5%–82% of patients and increases with disease duration.^5^, ^6^ The prevalence of cardiac involvements increases with age, disease duration, and the presence of peripheral arthritis.^4^ Nevertheless, aortic involvement is considered a rare but life‐threatening complication of AS, which mostly involves the aortic root and ascending aorta, that can lead to valvular insufficiency.^1^, ^4^ In this report, we present a case in which the patient, despite being young and symptomatic for only 1 year, had no evidence of peripheral arthritis, and was diagnosed with aortitis, an uncommon complication of AS.

## 2 CASE PRESENTATION

A 34‐year‐old man with no medical history presented to a medical facility with intermittent fever, myalgia, and polyarthralgia for 1 year. The symptoms initially appeared monthly but gradually the intervals between fevers were shortened to days. He also had a history of significant weight loss, night sweats, and nonspecific mechanical back pains but no respiratory symptoms, including cough, dyspnea, or hemoptysis, nor a history of contacting animals or eating unsanitary dairy. In addition, there was no history of skin or mucosal lesions or urinary symptoms. The patient was from a *Brucella*‐endemic region in the country, did not travel to malaria‐prone areas recently, and his fevers did not follow a fixed pattern. The drug history was acetaminophen and antibiotics, administered during his fever and pain periods. He had no allergic history, or substance or alcohol abuse history, and there was not any history of malignancy or similar symptoms in his close family members reported.

In the reported lab data, he had an elevated erythrocyte sedimentation rate (ESR) and C‐reactive protein (CRP), mild leukocytosis, and negative blood and urine cultures. Also, there was no pathological lymphadenopathy in the neck, chest and abdominal computed tomography (CT) scans. He did not have abdominal or chest pain during the fevers or a family history of familial Mediterranean fever (FMF). Still, with a preliminary diagnosis of FMF, colchicine was administered and FMF genetic testing was done, which was negative. The patient's symptoms had no improvement with colchicine, and macular rashes appeared on his back during treatment. Therefore, skin biopsy was done, which was nondiagnostic, and due to resolving the rashes after discontinuing colchicine, the drug reaction was considered the cause of skin rash.

The patient was referred to our hospital 12 months after the onset of symptoms due to the absence of an accurate diagnosis. On our first visit, he was a middle‐aged man with a low body mass index (BMI) and cachectic appearance. The vital signs were stable in physical examination, except for a 38.7 centigrade fever. Physical examination revealed no signs of skin lesions, including malar rash, and no mucosal and genital ulcer or aphthous, in addition to a normal fundoscopic examination. There was no lymphadenopathy or hepatosplenomegaly and the examination of joints, including peripheral and central, was unremarkable. He had 5/5 force in the distal and proximal of both lower and upper limbs. There was a limitation in the movements of the back in flexion and the Schuber borderline test revealed a measurement of 5 cm.

At the onset of the disease, the patient experienced mechanical back pain and polyarthralgia; however, over a relatively short period, the intensity of these symptoms escalated, and both polyarthralgia and back pain transitioned into an inflammatory state. This transition was particularly evident when we first evaluated the patient. It is noteworthy that the patient described the pain as inflammatory in nature from the onset of the disease.

Due to an undefined source of intermittent fevers a complete laboratory workup was performed (Table 1). Furthermore, to investigate infectious causes of fever of unknown origin (FUO), HIV ELISA, HBs‐Ag, and Anti HCV antibody were tested and reported negative. A peripheral blood smear done for malaria was also negative, the same as Wright, Coombs Wright, and 2ME.

**TABLE 1:** Laboratory and paraclinical findings of the case of ankylosing spondylitis with fever.

Laboratory test	Value	Normal range
White blood cell; (×10^9^/L)	10,800	4500–12,500
Hemoglobin; g/dL	15	12–16
Platelet count; μL	361,000	150,000–450,000
Mean corpuscular volume; fL	79	80–98
Blood urea nitrogen; mg/dL	12	7–20
Creatinine; mg/dL	0.9	0.5–1.3
Sodium; mEq/L	140	135–145
Potassium; mEq/L	3.9	3.5–5.5
Calcium; mg/dL	9.2	8.5–10.5
Albumin; g/dL	4	3.4–5.4
Magnesium; mEq/L	2.1	1.8–2.6
Venous blood gas		
pH	7.4	7.35–7.45
Partial pressure of carbon dioxide; mmHg	36	40
Bicarbonate; mEq/L	24	24
Interferon‐gamma release assays	Negative	‐
Rapid plasma regain	Negative	‐
Antinuclear antibody	12	40–55
Anti‐double‐stranded deoxyribonucleic acid antibody	0.1	<0.9
Rheumatoid factor; IU/ml	11	<20
Anti‐cyclic citrullinated peptide; U/mL	1.8	<25
Anti–Sjögren's syndrome‐related antigen A autoantibodies; U/mL	8.6	<20
Anti–Sjögren's syndrome‐related antigen B autoantibodies; U/mL	6	<20
Complement component 3; mg/dL	**191**	90–180
Complement component 4; mg/dL	**41**	10–40
Total Hemolytic Complement; U/mL	120	70–120
Cytoplasmic antineutrophil cytoplasmic antibodies	0.9	<10
Perinuclear antineutrophil cytoplasmic antibodies	1.4	<10
Erythrocyte sedimentation rate; mm/h	**110**	0–25
C‐reactive protein; mg/L	**96**	<5
Ferritin; ng/mL	169	30–400
Triglycerides; mg/dL	141	<150
Aspartate aminotransferase; IU/L	18	<31
Alanine transaminase; IU/L	22	<31
Alkaline phosphatase; IU/L	161	80–300
Urinalysis	Specific Gravity = 1030 Urine PH = 5 No casts or red blood cells	‐

*Note*: Bold values indicate abnormal findings based on reference level.

An echocardiography was performed, showing normal ejection fraction and normal pulmonary artery pressure with no significant valvular disease or signs of vegetation, and a neurology consult was done, requesting a lumbar puncture, which was negative for chronic meningoencephalitis. In order to conduct a malignancy workup, a neck, chest, abdominal and pelvic CT scan with intravenous contrast was done. There was no pathological lymphadenopathy or other signs of malignancy, and serum and urine protein electrophoresis had no diagnostic feature. Endo‐colonoscopy had no pathological finding, and according to the hemato‐oncology consult, bone marrow aspiration biopsy would not be diagnostic due to normal complete blood cell count. For investigating the rheumatological causes, there were no diagnostic symptoms or signs of organ involvement. Lab data, as mentioned, were leading nowhere, and seronegative diseases were only probable. Lastly, due to nonspecific back pains, a lumbosacral and pelvic x‐ray was performed which was unremarkable. Based on the radiography results, unilateral sacroiliitis, and the endemicity of Brucella and tuberculosis, we conducted a biopsy to rule out infectious causes, which was negative. Based on the negative biopsy and unremarkable infectious‐related laboratory results, a sacroiliac magnetic resonance imaging (MRI) was done, showing signs of inflammation with no erosion. A positron emission tomography (PET) scan was ordered to diagnose the underlying probable malignancies, and a human leukocyte antigen B27 (HLA‐B27) test was requested to help diagnose probable AS, which was negative.

**FIGURE 1:**
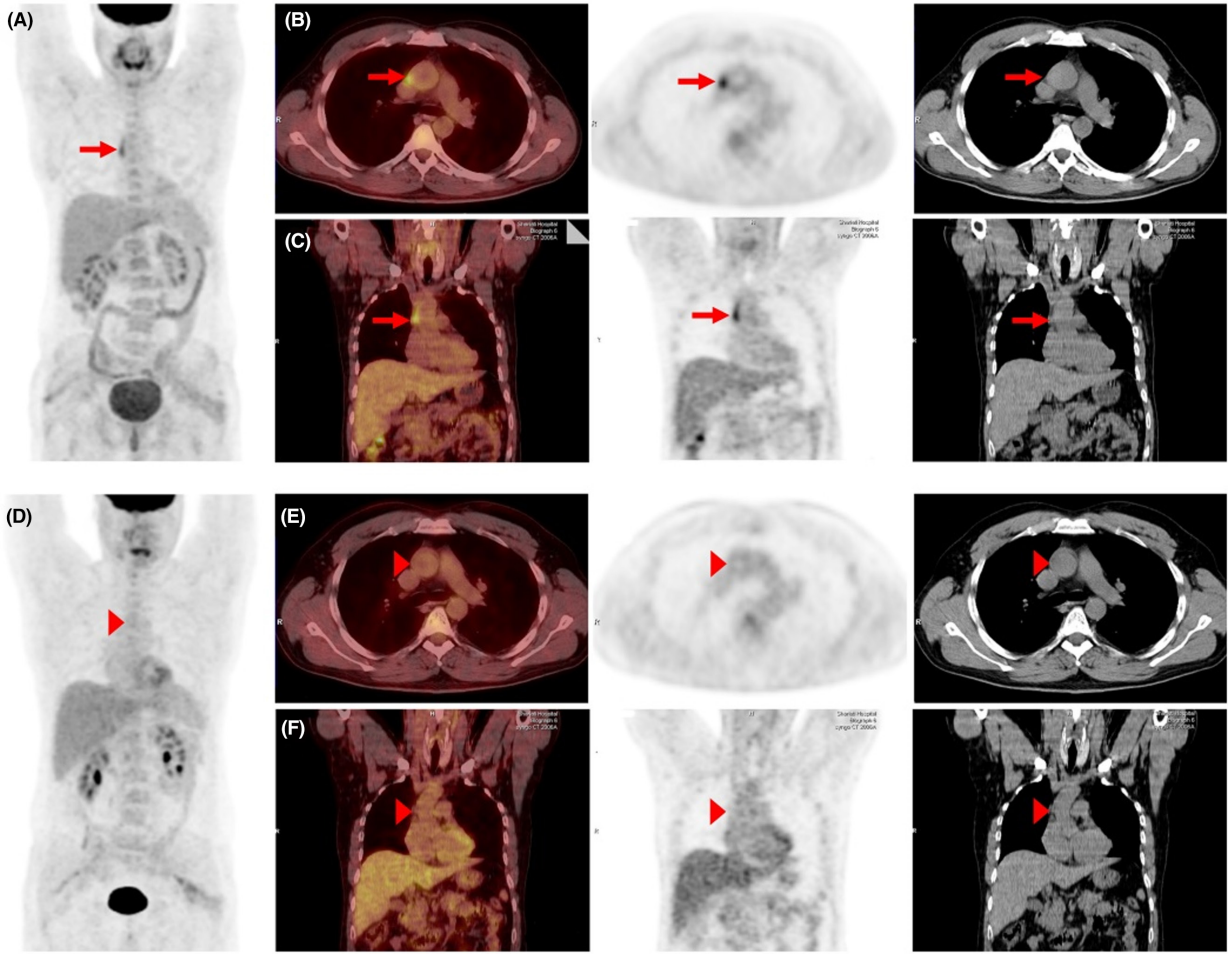
Fluorine‐18 fluorodeoxyglucose positron emission tomography/computed tomography images at the initial diagnosis (A–C), and follow‐up study 4 months after therapy (D–F); maximum‐intensity projection (A), transaxial (B), and coronal (C) images show a linear metabolic activity in the right lateral aspect of the proximal ascending.

The patient was discharged with 400 mg celecoxib as a probable AS case and attended a monthly follow‐up. In the first follow‐up, 4 weeks from the second infliximab injection, the patient stated that his pains and fevers resolved by about 30%, but in lab data, he still had elevated ESR and CRP. A PET scan revealed inflammation in ascending aorta compatible with aortitis, with no other abnormal uptake (Figure 1). The patient was finally diagnosed with spondylitis ankylosing with aortitis, and treatment was started with infliximab 300 mg for three sessions at 0, 2, and 6 weeks and 30 mg of prednisolone. During the patient's follow‐up after 4 months, his pain and fever gradually and entirely resolved, and ESR and CRP decreased significantly. The prednisolone was tapered to 10 mg, and non‐steroidal anti‐inflammatory (NSAID) drugs use continued. The PET SCAN was repeated after the third dose of infliximab and 4 months after the initiation of treatment, showing no inflammation (Figure 1).

## 3 DISCUSSION

Aortitis is a rare but serious complication associated with AS, and its recognition is crucial for timely intervention. It is characterized by chronic inflammation of the aortic wall, which involves the aortic root and ascending aorta leading to valvular insufficiency and conduction abnormalities when extended to the interventricular septum, aneurysm formation, and potentially life‐threatening aortic dissection.^1^, ^4^ Diagnosing aortitis in the context of AS can be challenging due to the fact that symptoms of AS and the underlying systemic inflammation overlap. The distinction between mechanical and inflammatory pain can be less clear in the initial stages of the condition. Clinical suspicion and a thorough workup are the first steps. The evaluation typically involves laboratory tests, including markers of inflammation such as ESR and CRP. Advanced imaging techniques, such as CT or MRI, are essential for detecting signs of inflammation and structural abnormalities within the aorta.^7^ In this case, the patient's symptoms, along with elevated ESR and sacroiliac joint involvement, raised suspicion of underlying systemic inflammation. However, unlike most cases of AS with cardiovascular involvement, our patient developed aortitis during the early phases of his disease. The PET scan played a pivotal role in detecting aortitis, emphasizing the value of recruiting advanced imaging techniques to evaluate the extent of systemic involvement in AS.

The pathogenesis of aortitis in AS is related to the chronic systemic inflammation characteristic of the disease.^4^ One of the first pathophysiologic studies of cardiovascular manifestations in AS was done by Bulkley and Roberts, who studied autopsy findings in eight patients with AS. The result was aortic root dilatation with fibrous proliferation along the intima.^8^ Prolonged inflammation in AS leads to the infiltration of immune cells into the aortic wall, causing endarteritis around the aortic root and valve.^9^ Therefore, prompt diagnosis and treatment could decrease the chances of long‐term morbidity and mortality.

Treatment of AS involves a multidisciplinary approach aimed at managing the underlying inflammation, preventing complications, and improving the patient's quality of life. NSAIDs are mainly the first‐line therapy for controlling pain and also reducing inflammation in AS. In cases where NSAIDs alone are insufficient, corticosteroids may be prescribed to achieve better results. Additionally, biologic agents targeting tumor necrosis factor, such as infliximab, have shown promising results in managing AS.^10^ Also, Tam et al highlighted the importance of proper treatment, particularly stating that sulfasalazine at its optimal daily dose reduces the risk of cardiovascular diseases in patients with AS.^11^ Potentially, more intensive anti‐inflammatory treatment of active AS may avert rapidly developing cardiovascular complications, such as coronary artery stenosis.^12^ In our specific case, NSAIDs, prednisolone, and infliximab were initiated to target the inflammatory process. The treatment resulted in a major improvement in signs and symptoms. However, the optimal treatment approach for aortitis in AS is still evolving, and further research is warranted to establish standardized protocols and assess long‐term outcomes.

Although studies have elaborated on implanting electrocardiography and echocardiography evaluation as part of the routine management of patients with AS, these studies mostly focus on patients of older ages or long‐withstanding AS.^13^ Aortic involvement is a potentially life‐threatening complication that may occur both in the late and, more rarely, in early evolution phases of AS, and increases with age, disease duration, and the presence of peripheral arthritis.^14^ However, this was not the case in our report, in which we demonstrated that aortitis was diagnosed in a young male patient with a negative HLA‐B27. The presence of the HLA‐B27 is proven to be highly associated with AS. However, there has been no evidence, that HLA‐B27 increases the chances of cardiovascular complications in AS patients.^15^ In our study, the HLA‐B27 test was also reported negative, aligning with the previous studies, showing no relationship between the presence of HLA‐B27 and the manifestation of cardiovascular complications. We believe that our report could contribute to further examining the current screening and approaches in managing patients with AS, to avoid possible morbidity and mortalities in a young patient with fevers, constitutional symptoms, back pain, and elevated inflammation markers—a specific history of the back pain helps us to order the right diagnostic test which can further explain his fevers and weight loss. In this patient, SI joint imaging was done due to back pain which showed sacroiliitis which led to the diagnosis of axial spondyloarthritis. Given his high markers, weight loss and constitutional symptoms, and spondyloarthritides diagnosis, we believe imaging to rule out or screen for vasculitis would be the next ideal step. Current treatment and management approaches should take into consideration the possibility of aortic and cardiovascular involvement, even in young patients without a long‐lasting history of disease or symptoms.

## 4 CONCLUSION

This case study emphasizes the significance of considering rare but critical manifestations of AS, such as aortitis, which can lead to potentially life‐threatening complications when undiagnosed and untreated. The timely recognition and diagnosis of aortitis in AS patients are essential to initiate appropriate treatment and prevent adverse outcomes. Therefore, routine cardiac examinations along with a low threshold to screen for vascular involvement in an axial or non‐axial spondyloarthritides spondyloarthritis presenting with unexplained fevers and significant constitutional symptoms and elevated markers should be considered as part of the comprehensive assessment of patients with AS to facilitate early detection of potential cardiovascular involvement. However, further research is needed to deepen our understanding of these rare complications, establish standardized diagnostic and treatment protocols, and assess long‐term outcomes. Increased awareness among clinicians and ongoing efforts to optimize patient care is crucial in addressing the challenges associated with rare manifestations of AS.

## AUTHOR CONTRIBUTIONS

**Mahsa Mehdipour Dalivand:** Data curation; investigation. **Rezvan Abdolazimi:** Data curation; investigation. **Reyhaneh Manafi‐Farid:** Investigation; methodology. **Ahmadreza Jamshidi:** Data curation. **Kimia Kassaee:** Writing – original draft; writing – review and editing. **Sara Foolad:** Writing – original draft. **Majid Alikhani:** Conceptualization; investigation.

## FUNDING INFORMATION

No financial support was received for this report.

## CONFLICT OF INTEREST STATEMENT

The authors declare that they have no competing interests.

## ETHICS STATEMENT AND CONSENT TO PARTICIPATE

Written informed consent was obtained from the patients in our study. The purpose of this research was completely explained to the patients and they were assured that their information would be kept confidential by the researcher.

## CONSENT

Written informed consent was obtained from the patient for the publication of this report. A copy of the written consent is available for review by the Editor of this journal.

## Data Availability

All data regarding this study has been reported in the manuscript. Please contact the corresponding author if you are interested in any further information.
